# Cervical cancer screening and vaccination: knowledge, awareness, and attitude of female staff in a Nigerian University

**DOI:** 10.1186/s12905-023-02345-9

**Published:** 2023-05-03

**Authors:** Laofe Ogundipe, Tolulope Ojo, Tunrayo Oluwadare, Eniola Olayemi, Funmilayo Oluwafemi, Olawale Oni, Olasumbo Kukoyi, Edidiong Orok

**Affiliations:** 1 Department of Public health, College of Medicine and Health Sciences, Afe Babalola University, Ado-Ekiti, Nigeria; 2 Department of Clinical Pharmacy and Public Health, College of Pharmacy, Afe Babalola University, Ado-Ekiti, Nigeria

**Keywords:** Cervical cancer screening, Awareness, Knowledge, Attitude, Female workers, Nigeria

## Abstract

**Background:**

Poor knowledge and awareness of cervical cancer screening and vaccination are significant barriers to effective cervical cancer prevention in developing countries. Knowledge of cervical cancer and vaccination against cervical cancer remains low in Nigeria. The purpose of this study was to assess the knowledge, awareness, and attitude of female staff of Afe Babalola University towards cervical cancer screening and vaccinations.

**Methods:**

This study was a cross-sectional study conducted using a semi-structured questionnaire among female staff of Afe Babalola University, Ado-Ekiti, Ekiti State, Nigeria. The workers’ knowledge and awareness were assessed using yes and no questions while the attitude was assessed using Likert scale questions. The workers’ knowledge was ranked as good (≥ 50%) and poor (< 50%) while attitude was ranked as positive (≥ 50%) and negative (< 50%). The relationship between demographics, attitude and knowledge of cervical cancer screening and vaccination was carried out using the Chi-square test. Analyses were conducted using SPSS software version 20.

**Results:**

A total of 200 staff consented to participate in the study out of which 64% were married with mean age 32.81 ± 8.164 years. Majority (60.5%) of the participants knew the causes of cervical cancer while 7.5% strongly agreed that they do not see the need for cervical screening. Majority (63.5%) of the participants showed good knowledge while 46% had a positive attitude towards cervical cancer screening and vaccination.

**Conclusions:**

The study participants showed good knowledge and awareness but poor attitude towards cervical cancer screening and vaccinations. Interventions and continuous education are needed to improve the population’s attitude and eliminate misconceptions.

## Background

Cervical cancer is the fourth most frequent cancer in women worldwide, with 604 000 new cases and 342 000 deaths in 2020 [1]. In 2020, low- and middle-income nations accounted for over 90% of cervical cancer new cases and related deaths worldwide [2]. Cervical cancer death rate has also been reported to be 18 times higher in low income countries than in developed countries [3] and some nations in Sub-Saharan Africa, Latin America, and Asia have exceptionally high cervical cancer-related death rates [4, 5].

Cervical cancer is the second most frequent cancer among Nigerian women aged 15 to 44 with over 14,000 new diagnoses and more than 20 deaths daily [4, 6]. Also, Nigeria has one of the highest rates of human papillomavirus (HPV)-related diseases in Sub-Saharan Africa [7] and research findings suggest that HPV is highly prevalent among Nigerian women. According to Aminu et al., (2014) [8], Immunoglobulin G (IgG) antibodies against HPV had a prevalence above 40% among Nigerian women in the northern region. HPV infection rates in female outpatients in Southwest Nigeria have also been reported to range from 30.4 to 36.5% [9, 10].

A persistent infection with the sexually transmitted human papillomavirus (HPV) is the most common cause of cervical cancer [11]. HPV is responsible for 90–100% of cervical cancer cases in women, especially those under the age of 35 [12]. HPV is the most prevalent viral infection of the female reproductive system and the majority of sexually active women and men will become infected at some point in their lives, while some will become infected multiple times [1].

Comprehensive prevention and control strategies for cervical cancer have been proposed by the World Health Organisation in 2020 [13]. The strategies encompassed primary, secondary and tertiary prevention strategies such as community education, social mobilization, vaccination, screening, treatment and palliative care. Vaccination against HPV, pre-cancerous lesion screening and therapy, early identification and rapid treatment of invasive malignancies, and palliative care are established cost-effective options for addressing cervical cancer across the care continuum [14]. Expanded HPV vaccination coverage is expected to eradicate roughly 70% of cervical cancers globally, and cervical cancer screening performed by women in their 30 or 40 s could reduce the risk of cervical cancer by 25–30% [15]. Vaccination against HPV infection among adolescents before their first sexual experience is one of the most important cervical cancer preventive measures [16]. The provision of HPV vaccines in low- and middle-income countries is a key component of the global action plan to reduce cervical cancer prevalence [17]. The World Health Organization (WHO) recommends giving HPV vaccine to girls between the ages of 9 and 13 before sexual exposure because the vaccine is most effective if the girls have not already been infected with HPV [18]. Girls between 9 and 13 years of age should receive a two-dose HPV vaccine regimen with a 6-month interval between doses (0, 6 months), while women aged 16 to 26 years old can receive a three-dose regimen (0, 1, and 6 months or 0, 2, and 6 months), with cervical cancer screening still required after HPV vaccination [19]. Cervical screening can be done with one of three types of tests that are now accessible and commonly utilised namely HPV DNA testing, cytology-based Papanicolaou tests (Pap tests), and unaided visual inspection with acetic acid (VIA). However, there is a lack of public awareness of these tests, particularly in underdeveloped nations [20].

In contrast to low-income countries, the developed countries have seen a decline in the incidence and mortality of HPV infection and cervical cancer due to widespread availability and use of the HPV vaccine and cervical cancer screening [21, 22]. Low awareness and poor knowledge on cervical cancer screening and vaccination approaches has been identified as major barriers to effective cervical cancer prevention in developing countries [23].

Although the HPV vaccine was first introduced in Nigeria in 2009, the knowledge and vaccination against HPV as a preventive intervention against cervical cancer as well as vaccination uptake by the target demographic of young people, remains low [24].According to recent research, less than 15% of adolescent girls had got the HPV vaccine [25, 26] and about 10% of women had cervical cancer screenings [27, 28]. Similarly, Awodele et al., (2011) reported low cervical screening uptake among more than 150 Nigerian nurses, with 60% reporting having never been checked for cervical cancer [29]. Parents and caregivers of young people in Nigeria have exhibited a lack of understanding about cervical cancer prevention and screening for their children [30, 31]. The lack of such knowledge may have a negative impact on HPV vaccine acceptability and uptake. Furthermore, the low acceptance of cervical screening in Nigeria has been linked to barriers such as lack of awareness, insufficient knowledge of diseases and preventive treatments, insufficient spousal support, misperceptions, stigma, and cultural views resulting in poor outcomes and high mortality rates [22, 32–33].

The American Cancer Society recommends that women should begin cervical cancer screening at age 25 and undergo primary HPV testing every 5 years through age 65 or individuals should be screened every 5 years with co-testing (HPV testing combined with cytology) or cytology alone every 3 years [34]. Since, knowledge and awareness of cervical cancer, screening and vaccination is still low in Nigeria, it was pertinent to carry out this study.

This study was to assess the level of knowledge, awareness, and attitude regarding cervical cancer, its screening and vaccination among female staff at Afe Babalola University Ado-Ekiti.

## Methods

### Study area

The study was conducted among female workers of Afe Babalola University, Ado Ekiti (ABUAD). ABUAD is a private university in Ado-Ekiti, Ekiti State, Nigeria. It was established in 2009 by Afe Babalola, a lawyer and philanthropist. The programs offered include Science, Law, Engineering, Social and Management Sciences, Pharmacy, Medicine and Health Sciences, and Postgraduate Studies. It has a student population of about 8500 and a staff population of 1255 [35].

### Study Design and Duration

This study employed the cross-sectional survey design aimed at assessing the level of knowledge, awareness and attitude towards cervical cancer screening and vaccination among female staff of ABUAD, Ekiti State. This study was carried out from April to July 2021.

### Study Population

The target population for this study was all the female staff of Afe Babalola University, comprising teaching and non-teaching staff.

### Sample size determination

The sample size was calculated using Epi-Info for population survey [36].The Confidence interval was set at 95%. The Female staff population size of Afe Babalola is 366 and the expected frequency was set at 62.5% based on a similar study [37]. A confidence limit of 5% was used and contingency at 10% of calculated sample size was also considered.

$$ n=182+18.2=200.2$$

The calculated sample size was approximately 200 workers.

### Sampling technique

The simple random sampling technique was used. The sampling frame for the simple random sample was obtained from the registry. ABUAD has a total of 1255 staff, out of which 366 are female workers. The sample size was achieved by random selection through computer generated codes after assigning numbers to each staff.

### Instrument for data collection

The 34- item semi-structured adapted questionnaire was used for data collection in this study, designed according to the variables being tested in the study. Section A elicited the demographic data of the participants while section B assessed the level of knowledge and awareness of cervical cancer screening and vaccination among the respondents. Section C determined the attitude of the population towards cervical cancer screening and vaccination. A total of 15 dichotomised “yes and no” questions were used to assess the participants’ knowledge and awareness, while 13 Likert scale questions were used to assess their attitude towards cervical cancer screening and vaccination. A score of 1 was allocated to each correct knowledge question, while a zero score was allocated to each wrong answer. A score of 5 represented the highest rank while 1 represented the lowest rank for the Likert scale questions.

### Validity and reliability of the instrument

The research instrument was assessed for face validity by lecturers of the Department of Public Health, College of Medical Health Sciences, Afe Babalola University. Prior to the administration of the questionnaire, a pre-test was done a week before the actual data collection with 10 participants that have similar characteristics to the study population but were not included in the final data. The reliability coefficient was calculated to test for the internal consistencies of response using the Cronbach Alpha coefficient and to determine if the instrument is reliable for the study. A value of 0.72 was obtained.

### Data collection

This research questionnaire was self-administered, as the questionnaire was simple and clear for the participant questions. The questionnaires were handed out to selected staff in their respective colleges daily and only participants that met the inclusion criteria participated in the study. The questionnaire required an average of 10 min to complete.

### Data analysis

Data analysis was conducted using Statistical Package for Social Sciences (SPSS) version 20. The staff socio-demographics, knowledge, awareness and attitude towards cervical cancer screening and vaccination were summarized using frequency counts and percentages. Overall staff knowledge was categorised into good (≥ 50%) and poor knowledge (< 50%) while attitude was categorised into positive (≥ 50%) and negative (< 50%). The relationship between demographics, attitude and knowledge of cervical cancer screening and vaccination was carried out using the Pearson’s Chi-square test. All significant differences were set at p < 0.05.

### Ethical consideration

Research ethics approval was obtained from Afe Babalola University Health Research Ethics Committee (ABUADHREC). A written informed consent was obtained from all selected participants after thorough explanation of the research information. Confidentiality of each participant was ensured by exclusion of their names and other confidential details when participating in the study.

## Results

### Demographics of the study participants

A total of two hundred respondents consented to participate in this study, and they all filled and returned the study questionnaires, representing more than 100% response rate. The mean age of the participants was 32.81 ± 8.164 years, with 4.5% (9) within the age range of 50 years and above. More than 70% (140) of the participants were Yoruba, while two-thirds (124; 62%) of the participants were teaching staff. The majority of the participants (80.5%) were Christians, while 64% (128) were married. The participant demographics are summarised in Table 1.

### Knowledge and awareness of participants on cervical cancer screening and vaccination

About two-thirds (60.5%) of the participants knew the causes of cervical cancer, while less than half of the participants (47.5%) admitted that Human Papilloma Virus (HPV) is a sexually transmitted virus that can cause cervical cancer. More than 54% (109) of the workers admitted knowing that cervical cancer is preventable through the use of HPV vaccines, while less than one-third of the participants (29.5%) admitted that cervical cancer is only common among older women above the age of 35. Ninety (45%) workers admitted that using a condom when having sexual intercourse can reduce the chance of getting HPV, while 31% (62) admitted that HPV vaccine is usually given within a period of six months in three doses (Table 2). Overall, majority (63.5%; 127) of the participants showed good knowledge. One hundred and thirty-two workers (66.1%) stated that they were informed by health workers, while 8 (3.8%) were informed by newspaper/magazine. Less than 5% (9) reported that they got their information through television/radio while 8.3% (17), 26.9% (54) and 1.3% (3) got to know about cervical cancer from the internet, friends/peer and church respectively. There was a statistically significant association between religion (p = 0.002), but no significant association between marital status (p = 0.063) and knowledge of the participants on cervical screening and vaccination (Table 3).

**Table 1:** Socio-demographic characteristics of the female workers

Variable		Frequency (n = 200)	Percentage (%)
**Age**	20–29	72	36
	30–39	95	47.5
	40–49	24	12
	50 and above	9	4.5
**Ethnicity**	Yoruba	143	71.5
	Igbo	38	19
	Hausa	10	5
	Others	9	4.5
**Occupation**	Teaching Staff	124	62
	Non-teaching Staff	76	38
**College**	MHS	70	35
	Sciences	49	23
	SMS	66	33
	Law	14	7
	Engineering	4	2
**Religion**	Christianity	161	80.5
	Islam	33	16.5
	Other	6	3
**Marital Status**	Single	66	33
	Married	128	64
	Divorced/Separated	3	1.5
	Widow	3	1.5

**Table 2:** Knowledge of Workers on Cervical Cancer and Screening and Vaccination

	Statements	Correct (%)	Incorrect (%)
1	I know what causes cervical cancer	121 (60.5)	79 (29.5)
2	HPV is a sexually transmitted virus	95 (47.5)	105 (52.5)
3	Cervical cancer is preventable through the use of HPV vaccines	109 (54.5)	93 (45.5)
4	Using a condom when having sexual intercourse can reduce the chance of getting HPV	90 (45)	110 (55)
5	Cervical cancer is only common among older women above the age of 35	59 (29.5)	141 (70.5)
6	By having only one or few sexual partners one can reduce the chance of getting the HPV	82 (41)	118 (59)
7	Using birth control pills can prevent one from getting HPV thus preventing cervical cancer	26 (13)	174 (87)
8	The screening can only be done on women who are experiencing unusual symptoms	44 (22)	156 (78)
9	HPV is the leading cause of cervical cancer	100 (50)	100 (50)
10	HPV vaccine is usually given within a period of six months in three doses	62 (31)	138 (69)
	Good Knowledge (≥ 50%)	63.5%	
	Poor Knowledge (< 50%)	36.5%	

**Key**: HPV: Human Papilloma Virus

**Table 3:** Relationship between socio-demographic data, attitude and knowledge of Cervical cancer screening and vaccination

Characteristics	Level of knowledge		X^2^	P-value^a^
	Good Knowledge (%)	Poor Knowledge (%)		
**Age group**				
20–29	47(37)	25(12.5)	6.419	0.093
30–39	58(29)	37(18.5)		
40–49	13(6.5)	11(5.5)		
50 and above	9(4.5)	-		
**Religion**				
Christianity	108(54)	53(26.5)	12.613	0.002*
Islam	13(6.5)	20(10)		
Other	6(3)	-		
**Marital status**				
Single	44(22)	22(11)	7.284	0.063
Married	80(40)	48(24)		
Divorced/Separated	-	3(1.5)		
Widow	3(1.5)	-		
**Occupation**				
Academic staff	64	60	5.435	0.100
Non-academic staff	38	38		
**Attitude**				
Positive Attitude	74(37)	18(9)	21.081	< 0.001*
Negative Attitude	53(26.5)	55(27.5)		

Key: ^a^=Pearson Chi-Square Test; *=statistically significant values

The majority of the participants were aware of cervical cancer (79%, 158) and cervical cancer screening (60%, 120). More than half of the participants (55.5%, 111) were aware of HPV vaccination, while 50 (25%) participants reported that they have been vaccinated against HPV.

### Attitude towards cervical cancer screening and vaccination

During assessment of the attitude of the participants towards cervical cancer screening and vaccination, it was reported that 48.5% (97) of the workers strongly agreed that they feel uncomfortable with a male doctor performing the colposcopy/pap test and 7.5% (15) strongly agreed that they do not see the need for cervical screening since there are no early clear signs or symptoms. Sixty-eight staff (34%) strongly disagreed that they do not think that cervical cancer screening and HPV vaccine are necessary while 43.5% (87) of the participants agreed that Pap test is very expensive. Less than 25% (41) strongly agreed that they feel uneasy talking about cancer while 16.5% (33) and 30% (60) workers stated that they don’t think there is any screening site in the primary health care facility close by and that they are scared of a cancer diagnosis and treatment respectively. Overall, less than half of the participants (46%) had a positive attitude towards cervical cancer screening and vaccination. The participant attitude findings towards cervical cancer screening and vaccination are summarised in Table 4. There was a significant relationship between knowledge and attitude of participant (p < 0.001) towards cervical cancer screening and vaccination (Table 3).

**Table 4:** Workers’ attitude towards Cervical cancer screening and vaccination

S/N	Statement	Responses (%)			
		SA	A	D	SD
1	I feel uncomfortable with a male doctor performing the colposcopy/pap test	97 (48.5%)	51 (25.5%)	39 (19.5%)	13 (6.5%)
2	I do not see the need for cervical screening since there is no sign or symptom	15 (7.5%)	80 (40%)	71 (35.5%)	34 (17%)
3	I do not see the need for HPV vaccination since there is no sign or symptom	14 (7%)	51 (25.5%)	93 (46.5%)	42 (21%)
4	I don’t think the cervical cancer screening and HPV vaccine are necessary	19 (9.5%)	38 (19%)	75 (37.5%)	68 (34%)
5	I think the pap test is very expensive	35 (17.5%)	87 (43.5%)	55 (27.5%)	23 (11.5%)
6	I think the vaccination is very expensive	30 (15%)	80 (40%)	63 (31.5%)	27 (13.5%)
7	I feel uneasy talking about cancer	41 (20.5%)	57 (28.5%)	77 (38.5%)	25 (12.5%)
8	I don’t think there is any screening site in the primary health care faculty close to me	33 (16.5%)	76 (38%)	82 (41%)	9 (4.5%)
9	I am too busy to find time for cervical cancer screening	36 (18%)	60 (30%)	91 (45.5%)	13 (6.5%)
10	It is against my religious belief	10 (5%)	20 (10%)	119 (59.5%)	51 (25.5%
11	It is against my cultural value	20 (10%)	13 (6.5%)	104 (52%)	63 (31.5%)
12	I don’t think my partner would allow me to participate in the screening	11 (5.5%)	19 (9.5%)	107 (53.5%)	63 (31.5%)
13	I am scared of a cancer diagnosis and treatment	41 (20.5%)	60 (30%)	61 (30.5%)	38 (19%)
	**Positive Attitude**	**92 (46%)**			
	**Negative Attitude**	**108 (54%)**			

Key: SA: Strongly Agree = 5; A: Agree = 4; D: Disagree = 2; SD: Strongly Disagree = 1

## Discussion

This study revealed that majority of the participants were aware of cervical cancer, cervical cancer screening and the Human Papilloma Virus. This finding is in contrast with a study in Ibadan, Nigeria, where all the participants were not aware of cervical cancer and screening for cervical cancer [38]. Similar findings were reported in Gondar town, Ethiopia in which 65.1% claimed to have heard of cervical cancer while 21.4% and 18.6% reported to have heard of cervical cancer screening and Human Papilloma Virus respectively [39].

This difference in awareness could be due to difference in study population in which majority of the respondents were teaching staff while an undergraduate population and participants having educational qualification less than university level were used in the study done in Ethiopia and Ibadan, Nigeria respectively. Half of the participants were aware of HPV vaccination, while one-fourth of the participants had been vaccinated. This is in contrast to findings from a study conducted in Lagos, which showed that less than 2% of the participants had heard of HPV vaccination and none had been vaccinated [40]. This difference might be due to the lack of health education programs regarding cervical cancer vaccination in the urban slum, which is believed to be the problem of most developing countries [41]. Certain strategies have been suggested in literature including the use of nomograms in the sensitization of women [42] and also showing the women results of outcomes from women with cervical cancer as a way of making them realise why preventive behaviour is important [43]. The level of awareness and knowledge of cervical cancer, its aetiology, screening as well as HPV vaccination in women can greatly influence their participation in screening and vaccination programs [44].

Health workers were reported as the major source of information about cervical cancer by the study participants, followed by friends, television/radio, and the internet. This is quite dissimilar to a study done in northern Nigeria, where media and workshops were highlighted as major information sources [45]. Also, in China 35.2% of participants reported to have heard of cervical cancer from friends, 33.9% responded that they heard of cervical cancer through television, and 15.5% by other means [46]. The difference might be due to differences in study population between the two studies in Nigeria and differences in media coverage, health service access and utilization levels between the two countries. The source of information can also explain the good awareness shown by the study participants. Healthcare professionals with adequate knowledge can play a huge role in influencing the beliefs and practices of the public positively by raising awareness [47].

Less than half of the participants admitted that Human Papilloma Virus (HPV) is a sexually transmitted virus. This is contrary to the study done in Lagos, where 76.2% agreed that Human Papilloma Virus (HPV) is a sexually transmitted virus [48]. This difference might again emerge from lack of insufficient health education programs regarding cervical cancer.

The success of cervical cancer prevention and control is largely dependent on cervical cancer screening programmes and human papillomavirus vaccinations, according to the WHO [49]. In this study, more than 50% of the participants admitted knowing that cervical cancer is preventable. This finding is consistent with the study done by Mengesha et al. (2020) in which 78% of the participant agreed that cervical cancer is preventable [39]. More efforts should be put into developing more cervical cancer screening and vaccination programmes and centres to achieve adequate cervical cancer prevention and control.

Majority of the study participants showed good knowledge of cervical cancer screening and vaccinations. This is in contrast with findings in Nigeria [40] as well as other developing countries where the women had poor level of knowledge of cervical cancer and its prevention [50, 51]. There was also a statistically significant association between participant knowledge and attitude towards cervical cancer screening and vaccination, which is similar to studies carried out in low income countries where a direct relationship between women’s knowledge and attitudes about cervical cancer and its prevention has been documented [52, 53]. The lack of information and participation in vaccination and screening has been shown to inevitably lead to an increase in the incidence of cervical cancer [54], Most cervical cancers are diagnosed at an advanced stage due to a lack of knowledge about screening procedures, risk factors, and symptoms. This could lead to the eventual use of invasive treatments such as surgery and treatment-related morbidity and complications in early stage disease [55, 56].

Regarding the assessment of study participants’ attitude, more than 50% of the participants reported that they feel uncomfortable with a male doctor performing the colposcopy/pap test. This is similar to the study done by Tavafian (2012) where 45.2% of the respondents agreed that lack of female screeners in health facilities contribute to not screening [57]. This may be as a result of religious reasons. Also, majority disagreed that they do not see the need for cervical screening since there is no sign or symptom and that they don’t think the cervical cancer screening and HPV vaccine are necessary. Majority of the workers also agreed that they are scared of a cancer diagnosis and treatment. This is in agreement with a study done by Malhotra and colleagues where 32.78% agreed that there is need for cervical screening since there is no sign or symptom, and 8.61% agreed that cervical cancer screening and HPV vaccine are not necessary [58]. Similarly, results of a study in Nigeria by Ifediora and Azuike (2018) reported that 24% of the respondents agreed they worry about the findings of the screening, 15.2% of the respondents agreed they would be too busy to take cervical screening, and 12.1% of the respondents agreed that screening is not necessary [59]. Majority of the participants of this study showed negative attitude towards cervical cancer screening and vaccination which is similar, though lower, than what was obtained from a study carried out in Qatar which revealed that over 60% had a poor attitude towards prevention and screening test of cervical cancer [50]. This finding indicates that despite the higher level of women’s knowledge regarding cervical cancer and its prevention, the majority still showed somewhat poor attitude. This could be due to influence of other factors that could impact health-seeking behaviours apart from health-related knowledge [60]. Further studies should be done to identify other factors that can influence the attitude of females towards cervical cancer screening and vaccinations, and interventions should also be put in place to combat these factors.

## Limitations

This research assessed the awareness, knowledge and attitude towards cervical cancer among the female workers within one institution in Nigeria which could imply that the findings are not generalizable to other institutions in Nigeria. However, the findings have provided valuable insight that could be built upon in further studies. Also, parity of the respondents as well as differences in knowledge and attitude based on parity was not assessed in this study. This could be an explorable aspect in future studies.

## Conclusion

The study participants showed good knowledge and awareness, but poor attitude towards cervical cancer screening and vaccination. Strategies such as comprehensive health education on the utilization of cervical cancer screening and vaccination achievable through community health services programs and campaigns should be encouraged to improve the population’s attitude and eliminate misconceptions.

## Data Availability

Data material used in this study was confidential and will be made available by the corresponding authors on request.

## Abbreviations

HPV: Human Papilloma Virus
IgG: Immunoglobulin G
VIA: Visual Inspection with Acetic Acid
WHO: World Health Organisation
HIV: Human Immunodeficiency Virus
Pap Test: Papanicolaou Tests
ABUAD: Afe Babalola University, Ado-Ekiti
ABUADHREC: Afe Babalola University Health Research Ethics Committee
SPSS: Statistical Package for Social Sciences
